# Pathogenesis and Clinical Characteristics of Hereditary Arrhythmia Diseases

**DOI:** 10.3390/genes15111368

**Published:** 2024-10-24

**Authors:** Shuang Guo, Lingfeng Zha

**Affiliations:** 1 Department of Vascular Surgery, Beijing Tsinghua Changgung Hospital, School of Clinical Medicine, Tsinghua University, Beijing 102218, China; gsa03649@btch.edu.cn; 2 Department of Cardiology, Union Hospital, Tongji Medical College, Huazhong University of Science and Technology, Wuhan 430022, China; 3 Hubei Key Laboratory of Biological Targeted Therapy, Union Hospital, Tongji Medical College, Huazhong University of Science and Technology, Wuhan 430022, China; 4 Hubei Provincial Engineering Research Center of Immunological Diagnosis and Therapy for Cardiovascular Diseases, Union Hospital, Tongji Medical College, Huazhong University of Science and Technology, Wuhan 430022, China

**Keywords:** arrhythmia diseases, heredity, clinical characteristics, pathogenesis

## Abstract

Hereditary arrhythmias, as a class of cardiac electrophysiologic abnormalities caused mainly by genetic mutations, have gradually become one of the most important causes of sudden cardiac death in recent years. With the continuous development of genetics and molecular biology techniques, the study of inherited arrhythmias has made remarkable progress in the past few decades. More and more disease-causing genes are being identified, and there have been advances in the application of genetic testing for disease screening in individuals with disease and their family members. Determining more refined disease prevention strategies and therapeutic regimens that are tailored to the genetic characteristics and molecular pathogenesis of different groups or individuals forms the basis of individualized treatment. Understanding advances in the study of inherited arrhythmias provides important clues to better understand their pathogenesis and clinical features. This article provides a review of the pathophysiologic alterations caused by genetic variants and their relationship to disease phenotypes, including mainly cardiac ion channelopathies and cardiac conduction disorders.

## 1. Introduction

Hereditary arrhythmias are a group of electrophysiological abnormalities of the heart caused primarily by mutations in ion channel genes involved in regulating the action potential of the heart, including long QT syndrome (LQTS), short QT syndrome (SQTS), Brugada syndrome (BrS), catecholaminergic polymorphic ventricular tachycardia (CPVT), etc., ion channel disease, and cardiac conduction disease [1]. The common feature of these diseases is an irregular heartbeat with a wide range of clinical manifestations, from mild palpitations and fainting to severe sudden death. In recent years, hereditary arrhythmia has gradually become one of the most important causes of sudden cardiac death, the average age of sudden cardiac death is significantly lower than other causes, and there is a significant familial aggregation [2]. These diseases pose a great threat to the life and health of patients, but also impose a heavy burden on their families and society. Similarly, due to the complex phenotype of such diseases, high fatality rate, and great harm, it also poses a huge challenge to the diagnosis and treatment of clinicians [3]. Therefore, the in-depth study of hereditary arrhythmia is particularly important.

Over the past few decades, there have been significant advances in the study of hereditary arrhythmias. With the continuous development of genetics and molecular biology technology, more and more pathogenic genes have been identified, providing us with important clues to understand the pathogenesis of these diseases [4]. The discovery of pathogenic and susceptible genes related to arrhythmias and the establishment of molecular models have opened up a new direction for the treatment of arrhythmias. However, there remains considerable room for improvement in our understanding and treatment of hereditary arrhythmias. Despite advances in the accurate assignment of pathogenicity, a large number of rare mutations have been classified as having uncertain significance. In a word, our understanding of hereditary arrhythmias is far from complete.

Hereditary arrhythmia is a single gene disease, treatment options are few, and, theoretically speaking, gene therapy is the most ideal choice. Gene therapy can deliver genetic material into cells for purposes such as gene replacement, allele-specific silencing, modulation of signaling pathways, splicing regulation, and genome editing, utilizing viral vectors, oligonucleotides, and modified mRNAs [5]. The remarkable success of several gene therapy trials has reignited enthusiasm for the potential of gene therapy. The United States’ Food and Drug Administration (FDA) approved the first commercially available gene therapy, the adeno-associated virus treatment voretigene neparvovec-rzyl (Luxturna) in 2017 [6]. Since then, a variety of clinical trials related to gene therapy have surged, reaching a total of 11,683 studies as of this year. The advent of gene therapy technology has given researchers more tools to explore treatments for these diseases [7]. However, currently, gene therapy for hereditary arrhythmias remains in the preclinical stage due to challenges such as insufficient transduction efficiency in humans, ensuring safety at effective doses, potential off-target effects, the risk of inducing pro-arrhythmia, limited durability of the treatment, and the lack of reversibility [5]. For example, the targeted silencing of the ryanodine receptor 2 (*RYR2*) gene mutation can reduce the expression level of the R4496C mutant RYR2 protein, increase the wild-type expression level, and reduce the incidence of ventricular arrhythmia induced by isoproterenol [8].

Despite great progress, there are still many unanswered questions waiting for scientists to explore. To better understand hereditary arrhythmias, we need to delve deeper into their genetic basis, pathogenesis, clinical manifestations, and treatment. This review will provide an overview of the research progress in these areas and analyze the current problems and challenges. By systematically collating and summarizing the existing literature, we can provide valuable references for future research and clinical practice, and further promote the development of the field of hereditary arrhythmia.

## 2. Cardiac Ion Channel Disease

Ion channels are protein channels in the cell membrane of the heart that control the flow of ions inside and outside the cell. Different types of ion channels are responsible for regulating different ion flows that affect the electrical activity of the heart. For example, the opening of sodium channels leads to a rapid influx of sodium ions, triggering the depolarization of cardiomyocytes and thus the cardiac process. The opening of calcium channels causes calcium ions to flow in and promote myocardial contraction. The opening of potassium channels leads to an outflow of potassium ions, repolarizing the heart muscle cells in preparation for the start of the next cardiac cycle. In addition, receptors on heart cells also play an important role. These receptors can receive signals from external substances and regulate the electrical activity of the heart by activating ion channels. For example, epinephrine can activate adenylate cyclase by binding to the β1-adrenergic receptor on heart cells, increasing the level of cyclic adenosine phosphate (cAMP) in the cell, thereby promoting the opening of sodium ion channels, increasing the rate of depolarization of cardiomyocytes, and enhancing the contractility of the heart. Ion channels and receptors play a crucial role in maintaining normal cardiac electrical activity by regulating the depolarization and repolarization processes of heart cell membranes and the contraction processes of heart muscle cells [9]. The abnormal function of any ion channel or receptor can lead to arrhythmia or other heart-related diseases [10]. Mutations in genes encoding sodium channels, such as *SCN5A*, *SCN1B*, *SCN2B*, *SCN3B*, *SCN4B*, *GPD1L*, *RANGRF*, and *SCN10A*, can disrupt sodium channels and cause abnormal sodium current flow, triggering ventricular arrhythmias. Mutations in the genes encoding calcium channels, such as *CACNA1C*, *CACNA1D*, *CACNB2*, *CACNA2D*, and *CACNG*, can make calcium channels disordered, calcium current flow abnormal, and lead to ventricular arrhythmia. Although these genetic mutations come from different genes, they may result in the same phenotype by affecting the function and action of ion channels. For example, CPVT-related mutations are primarily responsible for the dysregulation of calcium release from RYR2, while the mutation that causes LQT is mainly due to the weakening of calcium binding and the complete cancellation of calcium dependent inactivation, which prolong the action potential [11]. Later, we will describe in detail how these genetic mutations lead to these phenotypes.

A class of disease caused by the ion channel dysfunction of cardiomyocytes is called cardiac ion channel disease, most of which have special electrocardiogram (ECG) manifestations and are clinically characterized by malignant ventricular arrhythmia and sudden death, and are not accompanied by cardiac structural and anatomical abnormalities [10]. They can be divided into inherited and acquired. Inherited ion channel disease is mainly caused by the abnormal gene encoding of an ion channel subunit (α or β subunit) or channel regulatory protein, and the main phenotypes are familial LQTS, BrS, CPVT, familial SQTS, and other conduction defects [12]. Acquired ion channel disease may be caused by drug exposure, immunoglobulins, toxins that regulate ion channel function, or changes in the expression and/or regulation of ion channel proteins on the basis of a primary disease (e.g., heart failure).

### 2.1. Familial Long QT Syndrome

LQTS is the most common inherited ion channel disease, with a prevalence of about 1:2000, and is characterized by prolonged QT interval, abnormal T-wave, and Torsades de pointes (TdP) [13]. TdP can often end on its own, resulting in syncope, which is the most common symptom in patients with LQTS. LQTS is mostly inherited in an autosomal dominant manner, such as Romano–Ward syndrome (RWS), which is characterized by syncope [14]; Andersen Tawil syndrome (ATS), characterized by periodic muscle paralysis and abnormal hand and facial development [15]; Timothy syndrome, characterized by abnormal development of the heart, hands, face, and nerves [16]. Jervell–Lange–Nielsen syndrome (JLNS), which is associated with sensorineural deafness, is an exception, and is inherited in an autosomal recessive manner [17].

The 18 genes currently reported to cause LQTS encode cardiac ion channel subunits or proteins involved in regulating the ion current (Table 1). These gene mutations lead to (1) inactivation of potassium channels, weakening of slow delayed rectifier current (I_Ks_) and rapid delayed rectifier current (I_Kr_), and prolonged action potential phase 3; (2) enhancement function of sodium and L-type calcium channels, the inward current I_Na_ and I_Ca_ are sustained, and the 0, 1, and 2 phases of action potential are prolonged. This ion dysfunction leads to prolonged cardiac action potential and the susceptibility of cardiomyocytes to early afterdepolarization (EAD), which induces TdP and other ventricular arrhythmias, thereby prolonging the QT interval [18,19]; (3) intracellular calcium overload leads to delayed afterdepolarizations (DAD) after action potential repolarization, resulting in prolonged action potential [20]. About 70% of these variants are missense mutations, 15% are frameshift mutations, and in-frame deletion, nonsense mutation, and splice site mutation account for 3–6%, respectively. Different types of mutations or different mutation sites in the same gene can lead to different diseases (Table 1). The latest study found that *ALG10B* encoding α-1, 2-glucose-transferase B (ALG10B) protein is a new LQTS susceptibility gene, and the p.G6S mutant of *ALG10B* down-regulates ALG10B, resulting in human ether-a-go-go-related gene (*HERG*) transport defects and prolonged action potential duration [21].

### 2.2. Familial Short QT Syndrome

SQTS is a very rare autosomal dominant arrhythmia characterized by short QTc intervals on the electrocardiogram, with syncope and various types of arrhythmias, including atrial fibrillation (AF), ventricular fibrillation, supraventricular tachycardia, and pleomorphic ventricular tachycardia [22]. Due to malignant ventricular arrhythmias, SQTS are associated with a higher risk of syncope or sudden death than LQTS. Genetic studies have shown that the pathogenesis of SQTS is related to abnormal cardiac ion channels that regulate the cardiac action potential (Table 1) [23], such as (1) functional gain mutations in the voltage-gated potassium channel subunit coding genes *KCNH2*, *KCNJ2*, and *KCNQ1*, resulting in shortened phase 3 repolarization; (2) loss-of-function mutations in the voltage-gated calcium channel subunit coding gene *CACNA2D1*, leading to a shortened QT interval, manifested as SQTS, and phenotype overlap with BrS [24]. In addition to some mutations in the above four genes that have pathogenic effects on SQTS, three other genes reported in the literature have possible pathogenic mutations of SQTS or mutations of uncertain importance, namely the voltage-gated calcium channel subunit coding genes *CACNA1C* [25] and *CACNB2* [26], and the anion exchange protein coding gene *SLC4A3* [27]. The *SLC4A3* mutation slows down Cl^−^/HCO_3_^−^ exchange, and the increase in pH and decrease in Cl^−^ shorten the duration of the action potential, thereby shortening the QT interval.

**Table 1:** Pathogenic genes and pathogenic mechanisms of LQTS and SQTS.

Gene	Protein	Chromosomal Location	Pathogenic Mechanism and LQTS/SQTS		Other Related Diseases
*AKAP9* [28]	A-kinase anchor protein 9	7q21.2	↑I_Ks_	RWS	BrS
*ANK2* [29]	Ankyrin-2	4q25-q26	↑I_Na_ ↑I_Ca_	RWS	-
*CACNA1C* [30]	Voltage-dependent L-type calcium channel subunit α-1C	12p13.33	↑I_Ca_	Timothy syndrome	BrS
*CACNA2D1* [31]	Voltage-dependent calcium channel subunit α-2/delta-1	7q21.11	↓I_Ca_	SQTS	BrS
*CALM1* [32,33]	Calmodulin-1	14q32.11	Multiple channel	RWS	CPVT
CALM2 [32,33]	Calmodulin-2	2p21	Multiple channel	RWS	BrS, CPVT
*CAV3* [34]	Caveolin-3	3p25.3	↑I_Na_	RWS	-
*KCNE1* [35]	Potassium voltage-gated channel subfamily E member 1	21q22.12	↓I_Ks_	JLNS/RWS	AF
*KCNE2* [36]	Potassium voltage-gated channel subfamily E member 2	21q22.11	↓I_Kr_	RWS	AF
*KCNH2* [37,38]	Potassium voltage-gated channel subfamily H member 2	7q36.1	↓I_Kr_	RWS	-
			↑I_Kr_	SQTS	-
*KCNJ2* [38,39]	Inward rectifier potassium channel 2	17q24.3	↓I_K1_	ATS	AF
			↑I_K1_	SQTS	-
*KCNJ5* [40]	G protein-activated inward rectifier potassium channel 4	11q24.3	↓I_KACh_	ATS/RWS	-
*KCNQ1* [35,38,41]	Potassium voltage-gated channel subfamily KQT member 1	11p15.5-p15.4	↓I_Ks_	JLNS/RWS	AF
			↑I_Ks_	SQTS	-
*NOS1AP* [42]	Carboxyl-terminal PDZ ligand of neuronal nitric oxide synthase protein	1q23.3	↑I_Ca_↓I_Kr_	RWS	-
*SCN4B* [43]	Sodium channel subunit β-4	11q23.3	↑I_Na_	RWS	AF
*SCN5A* [44]	Sodium channel protein type 5 subunit α	3p22.2	↑I_Na_	RWS	BrS, AF, AS, SSS, spontaneous ventricular fibrillation, PCCD
*SCN10A* [45]	Sodium channel protein type 10 subunit α	3p22.2	↑I_Na_	RWS	BrS
*SNTA1* [46]	α-1-syntrophin	20q11.21	↑I_Na_	RWS	-
*TRDN* [47]	Triadin	6q22.31	↑I_Ca_	RWS	CPVT

I_Ks_—slow delayed rectifier current; I_Kr_—rapid delayed rectifier current; I_K1_—inward rectifying potassium current; I_KACh_—potassium current activated by acetylcholine; I_Na_—voltage dependent sodium current; RWS—Romano–Ward syndrome; BrS—Brugada syndrome; SQTS—short QT syndrome; CPVT—catecholaminergic polymorphic ventricular tachycardia; JLNS—Jervell–Lange–Nielsen syndrome; AF—atrial fibrillation; ATS—Andersen Tawil syndrome; AS—atrial standstill; SSS—sick sinus syndrome, PCCD—progressive cardiac conduction defect. The gene comes from HGNC. The names of chromosome loci and loci come from OMIM. The protein comes from UniProt. Genetically related diseases come from Orphanet.

### 2.3. Catecholaminergic Polymorphic Ventricular Tachycardia

CPVT is a rare and fatal genetic arrhythmia, with 80% of patients having one or more syncope, and the prevalence is about 1:10,000 [48]. It is characterized by exercise- and tension-induced adrenergic ventricular tachycardia (VT), manifested as dizziness, syncope, and even sudden death, but without structural cardiac abnormalities. CPVT can be inherited in both autosomal dominant (*RYR2*, *CALM1*) and autosomal recessive (*CASQ2*, *TRDN*, *TECRL*) ways [49]. The most common pathogenic gene of CPVT is the aniline receptor *RYR2* encoding the cardiac sarcoplasmic reticulum Ca^2+^ release channel (about 55–65%), followed by the troponin coding gene *CASQ2* (about 2%). Pathogenic mutations caused by *CALM1*, *TRDN*, and *TECRL* are rare. *CALM2* and *CALM3* are candidate genes for CPVT (Table 2).

Arrhythmias in CPVT are caused by non-action potential triggered spontaneous calcium release, which starts with a local event of a single calcium release unit and spreads to neighboring calcium release units to generate whole-cell calcium waves. Calcium ions released by the plasma membrane activate sodium–calcium exchangers (Na^+^, Ca^2+^ exchanger, exchanger, NCX) cause one Ca^2+^ to exchange with three Na^+^ to produce a transient inward current (Iti), which produces transient membrane depolarization after completion of the action potential, that is DAD [50]. When the DAD amplitude reaches the voltage threshold for Na^+^ channel activation, an action potential is generated and propagated throughout the heart to produce pre-systolic beats. The ability of spontaneous calcium release to cause calcium waves is affected by the balance between the amount of Ca^2+^ in the sarcoplasmic reticulum and the concentration of Ca^2+^ that induces the release of Ca^2+^ in the sarcoplasmic reticulum (i.e., the sarcoplasmic reticulum calcium threshold) [51]. Whereas the RyR2 channel plays a key role in controlling the threshold, any situation that promotes the opening of the RyR2 channel lowers the sarcoplasmic reticulum threshold, thereby inducing spontaneous calcium release during β adrenergic stimulation, triggering DAD and triggering activity that leads to life-threatening arrhythmias.

Most *RYR2* mutations result in enhanced function, that is, increased calcium sensitivity, but the mechanism by which different mutations cause disease is different. There are three hypotheses as follows: (1) Helper protein FKBP prolyl isomerase 1B (FKBP1B) plays a key role in the pathogenesis. FKBP1B binds to RyR2 to stabilize the closed state of the channel, and mutations reduce the affinity of the RyR2 channel to FKBP1B, thus increasing RyR2’s sensitivity to cytoplasmic Ca^2+^. Phosphorylation of protein kinase A (PKA) promotes dissociation of FKBP1B, which leads to the spontaneous release of Ca^2+^ from the sarcoplasmic reticulum, inducing DAD and triggering activity [52,53]. This mechanism still lacks sufficient evidence, and there are certain disputes. (2) Mutations affect RyR2’s response to intracavicular and cytoplasmic Ca^2+^ concentrations, leaving RyR2 channels still open at low levels of Ca^2+^ and promoting spontaneous calcium release and DAD production by lowering the threshold of the sarcoplasmic reticulum [54]. (3) Partial mutations decompress the RyR2 central domain and enhance Ca^2+^ sensitivity, thus promoting spontaneous calcium release [55].

The pathogenesis of CPVT can be attributed to abnormal Ca^2+^ homeostasis.

The CASQ2 protein is the major Ca^2+^ reservoir in the cardiac sarcoplasmic reticulum and regulates RyR2 through triadin and adaptor proteins. When the sarcoplasmic reticulum Ca^2+^ level is low, CASQ2 protein binds to RyR2 to close the channel, otherwise the channel is open [56]. *CASQ2* mutations reduce CASQ2 levels by increasing sensitivity to proteolytic enzymes and attenuate the regulatory capacity of RyR2, thereby promoting spontaneous calcium release [57]. *TRDN* mutations regulate Ca^2+^ both by affecting CASQ2’s interaction with RyR2 and by making the protein susceptible to degradation [58,59]. Calmodulin gene mutations may affect intracellular Ca^2+^ homeostasis by altering its Ca^2+^ binding properties. TECRL is an endoplasmic reticulum protein that is highly expressed in the heart, the pathogenesis of which has not been elucidated, and may be related to the decreased NCX activity when mutated [60].

**Table 2:** Pathogenic genes of CPVT.

Gene	Protein	Chromosomal Location	Other Heart-Related Diseases
*RYR2* [52,53,54,55]	Ryanodine receptor 2	1q43	Isolated arrhythmia ventricular dysplasia
*CASQ2* [56]	Calsequestrin-2	1p13.1	-
*CALM1* [61]	Calmodulin-1	14q32.11	RWS
* *CALM2* [61,62]	Calmodulin-2	2p21	RWS, BrS
* *CALM3* [61]	Calmodulin-3	19q13.32	-
*TECRL* [60,63]	Trans-2,3-enoyl-CoA reductase-like	4q13.1	-
*TRDN* [58]	Triadin	6q22.31	RWS

BrS—Brugada syndrome; RWS—Romano–Ward syndrome. * Candidate gene. The gene comes from HGNC. The names of chromosome loci and loci come from OMIM. The protein comes from UniProt. Genetically related diseases come from Orphanet.

### 2.4. Brugada Syndrome

The prevalence of BrS is about 1–5:10,000, manifested by ST segment elevation on the ECG, which usually induces arrhythmia at night or in a state of high fever, and is mostly inherited in an autosomal dominant way, except for BrS caused by the *KCNE5* gene mutation, which is inherited in an X-linked way [64]. BrS may have ≥1 (V1, V2) ST segment elevation ≥ 2 mm in the right thoracic region of the ECG after intravenous injection of class I antiarrhythmic drugs, which is prone to ventricular tachyarrhythmia and sudden death. Currently, a total of 21 BrS-related pathogenic genes have been reported (Table 3) [65], among which the major susceptibility genes include *SCN5A*, *SCN10A*, *CACNA1C*, *CACNB2*, *CACNA2D1*, *KCNJ8*, and *TRPM*. At present, candidate genes that have no evidence to prove their pathogenic effects include *SCN2B*, *RANGRF*, *PKP2*, *KCNE5*, and *AKAP9*, and the rest are BrS pathogenic genes.

According to the repolarization hypothesis, the abnormal gene causes the right ventricular epicardial current balance to shift out, resulting in the abnormal repolarization and promoting the action potential phase 2 re-entry, and the close re-entry may lead to tachycardia or early ventricular fibrillation. The reduced inward sodium current and the increased outward current result in a transwall voltage gradient that is characterized by ST segment elevation on the ECG [66]. According to the depolarization hypothesis, the expression level and reserve of some channel proteins (such as SCN5A) in the outflow tract of the right ventricle of the adult heart are lower than that of the right ventricle [66]. The right ventricular outflow tract conduction slows down more significantly when the sodium current decreases due to the abnormal gene. Based on the underlying mechanism of ST segment elevation in regional transmural ischemia, depolarization of the right ventricular outflow tract delays abnormal current and manifests as ST segment elevation [67].

**Table 3:** Pathogenic genes and pathogenic mechanisms of BrS.

Gene	Protein	Chromosomal Location	Pathogenic Mechanism	Other Heart-Related Diseases
*CACNA1C*	Voltage-dependent L-type calcium channel subunit α-1C	12p13.33	↓I_Ca_	Timothy syndrome
*CACNA2D1* [68]	Voltage-dependent calcium channel subunit α-2/delta-1	7q21.11	↓I_Ca_	SQTS
*CACNB2* [69]	Voltage-dependent L-type calcium channel subunit β-2	10p12	↓I_Ca_	-
*CALM2* [70]	Calmodulin-2	2p21	↓I_Ca_	RWS, CPVT
*TRPM4* [71]	Transient receptor potential cation channel subfamily M member 4	19q13.3	↓I_Na_	PCCD
*SCN5A* [72]	Sodium channel protein type 5 subunit α	3p22.2	↓I_Na_	RWS, AF, AS, SSS, spontaneous ventricular fibrillation, PCCD
*SCN10A* [73]	Sodium channel protein type 10 subunit α	3p22.2	↓I_Na_	RWS
*SCN1B* [74]	Sodium channel subunit β-1	19q13.11	↓I_Na_	PCCD, AF
* *SCN2B* [75]	Sodium channel subunit β-2	11q23.3	↓I_Na_	AF
*SCN3B* [76]	Sodium channel regulatory subunit β-3	11q24.1	↓I_Na_	AF
*GPD1L* [77]	Glycerol-3-phosphate dehydrogenase 1-like protein	3p22.3	↓I_Na_	-
* *RANGRF* [78]	Ran guanine nucleotide release factor	17p13	↓I_Na_	-
*SLMAP* [79]	Sarcolemmal membrane-associated protein	3p14.3	↓I_Na_	-
* *PKP2* [80,81]	Plakophilin-2	12p11.21	↓I_Na_	Isolated arrhythmic ventricular dysplasia, left ventricular insufficiency
*KCND3* [82]	A-type voltage-gated potassium channel KCND3	1p13.2	↑I_t0_	-
*KCNE3* [83]	Potassium voltage-gated channel subfamily E member 3	11q13.4	↑I_t0_	-
* *KCNE5* [84]	Potassium voltage-gated channel subfamily E regulatory β subunit 5	Xq23	↑I_K,slow_	-
*KCNJ8* [85]	ATP-sensitive inward rectifier potassium channel 8	12p12.1	↑I_K-ATP_	-
*HCN4* [86]	Potassium/sodium hyperpolarization-activated cyclic nucleotide-gated channel 4	15q24.1	↓I_f_	SSS
*ABCC9* [87]	ATP-binding cassette subfamily C member 9	12p12.1	↑I_K-ATP_	AF, idiopathic dilated cardiomyopathy
* *AKAP9* [88]	A-kinase anchor protein 9	7q21.2	-	RWS

I_t0_—transient outward potassium current; I_K, slow_—slow transient outgoing potassium current; I_K-ATP_—ATP sensitive potassium current; I_Ca_—inward calcium current; I_Na_—inward sodium current; I_f_—inward pacemaker current formed by selective cation channels conducting Na^+^ and Ca2^+^; SQTS—short QT syndrome; RWS—Romano–Ward syndrome; CPVT—catecholaminergic polymorphic ventricular tachycardia; PCCD—progressive cardiac conduction defect; AF—atrial fibrillation; AS—atrial standstill; SSS—sick sinus syndrome. * Candidate gene. The gene comes from HGNC. The names of chromosome loci and loci come from OMIM. The protein comes from UniProt. Genetically related diseases come from Orphanet.

### 2.5. Familial Sick Sinus Syndrome (SSS)

SSS is a series of disorders with abnormal cardiac impulse formation and abnormal proliferation of the sinus node, and the prevalence is less than 1: 1 million [89]. SSS mainly affects the elderly and is clinically characterized by arrhythmias, including sinus bradycardia, sinus arrest, or alternate bradycardia and tachycardia. Early symptoms and signs are mild, but as the disease progresses, peripheral organ perfusion leads to progressively worse symptoms, including fainting, dizziness, and fatigue. SSS has both internal causes, such as the most common lymph node tissue degenerative fibrosis and ion channel dysfunction, sinus node remodeling, and external causes, such as autonomic dysfunction, increased vagal tone, metabolic disorders, drugs, toxins, and so on [90,91].

Most familial SSSs are autosomal dominant, but there is also an autosomal recessive inheritance of complex heterozygous *SCN5A* mutations. Familial SSS is mainly resulted from ion channel dysfunction caused by mutations in the following four genes: (1) mutation of *SCN5A*, encoding the pore forming α-subunit of the heart Na^+^ channel inactivates or significantly impairs the function of Na_V_1.5 channel, and Na_V_1.5 exists around the atrial muscle and the sinus node, so the myocardial excitability of the sinus node and the atrium is reduced, resulting in increased conduction time of the SA node, SA node conduction block, and bradycardia [92]; (2) *HCN4*, which encodes the hyperpolarized cyclic nucleotide-gated channel, is highly expressed in the sinoatrial node and lacks the nucleotide-binding domain after shortened mutation. As a result, the pacemaker current mediated by the HCN4 channel is insensitive to the increase in cAMP levels in cells [93], and the spontaneous release of Ca^2+^ driven pacemaker current by the sarcoplasmic reticulum is weakened [94]; (3) mutations in MYH6, the gene encoding α-myosin heavy chain, lead to sarcomere structural damage and atrial action potential conduction dysfunction, making individuals with pathogenic mutations susceptible to SSS [95]; (4) *DNAJB6*, which encodes the chaperone protein of the heat shock protein 40 family, is a novel SSS pathogenic gene with a unique expression pattern in the sinoatrial node pacemaker cell subpopulation, partially overlapping with the expression of HCN4, supporting the heterogeneity of cardiac pacemaker cells [96].

### 2.6. Familial Atrial Fibrillation

Familial AF is a rare autosomal dominant inherited arrhythmia characterized by abnormal atrial activation and irregular ventricular response in multiple members of a family [97]. They may be asymptomatic or have palpitations, dyspnea, and dizziness, often accompanied by dysrhythmia and cardiomyopathy. The occurrence of AF is related to many factors, such as comorbidity, heredity, gender, and lifestyle, while familial AF is caused by mutations in or near genes encoding voltage-gated potassium channel subunits, voltage-gated sodium channel subunits, transcription factors, and structural proteins (Table 4). The following summarizes 22 familial AF-related pathogenic genes reported so far, including 3 candidate genes.

The pathogenesis of the familial AF gene includes the following: (1) acquired potassium channel function mutations that lead to an enhanced potassium current (I_Kr_, I_Ks_, I_K1_, I_Kur_) function and a shortened action potential duration and atrial refractory period, thereby promoting AF [98].

(2) Potassium channel dysfunction mutations lead to a weakened potassium current (I_Kur_), and the extension of the atrial action potential and an effective refractory period (ERP) lead to an increased EAD tendency and increased AF sensitivity [99]. (3) A gain in sodium channel function mutation increases sodium current, fails repolarization, induces EAD and triggers activity, increases conduction velocity, and helps maintain fibrillation [100]. (4) A decreased or abnormal PITX2 expression reduces the expression of *NKX2-5*, and the *NKX2-5* mutation may promote AF production by over-activating the pacing current of the HCN4 channel [101]. Both NPPA and GJA5 are direct targets of the transcription factor Nkx2-5, and mutations in *NKX2-5* reduce their transcriptional activity [102]. (5) Mutations in *GJA5*, encoding cardiac gap junction protein (Connexin, Cx) 40, impair gap junction assembly or electrical coupling and increase AF susceptibility in patients [103]. Cx43 is hyperphosphorylated and translocated due to dysfunctional mutation of MYL4, which regulates channel permeability and promotes AF wave generation [104]. (6) When the *NPPA* encoding gene of natriuretic peptide precursor A is mutated, the cGMP level is up-regulated, the cAMP level and PKA activity are down-regulated, the cardiac sodium and calcium current is weakened, the rectified potassium current is enhanced, the action potential duration is shortened, and re-entrant atrial fibrillation is promoted [105]. (7) The mutation of *NUP155*, encoding nuclear porin reducing nuclear membrane permeability, changes nuclear localization, and inhibits nuclear Hsp70 mRNA and nuclear Hsp70 protein [106]. In addition, mutations of cardiac morphogenetic transcription factors *GATA4*, *GATA5*, *GATA6*, and *NKX2-6* alter transcriptional activity, which may affect AF through changes in the expression level of target genes [94].

**Table 4:** Pathogenic genes and pathogenic mechanisms of familial AF.

Gene	Protein	Chromosomal Location	Pathogenic Mechanism	Other Heart-Related Diseases
*KCNA5* [99]	Potassium voltage-gated channel subfamily A member 5	12p13.32	↑↓I_Kur_	-
*KCNJ2* [107]	Inward rectifier potassium channel 2	17q24.3	↑I_K1_	ATS, SQTS
*KCNQ1* [107,108]	Potassium voltage-gated channel subfamily KQT member 1	11p15.5-p15.4	↑I_Ks_	RWS, JLNS, SQTS
* *KCNE1* [108]	Potassium voltage-gated channel subfamily E member 1	21q22.12	↑I_Ks_	JLNS, RWS
*KCNE2* [107]	Potassium voltage-gated channel subfamily E member 2	21q22.11	↑I_Kr_	RWS
* *SCN1B*	Sodium channel subunit β-1	19q13.11	↑I_Na_	BrS, PCCD
*SCN2B*	Sodium channel subunit β-2	11q23.3	↑I_Na_	BrS
*SCN3B*	Sodium channel regulatory subunit β-3	11q24.1	↑I_Na_	BrS
*SCN4B*	Sodium channel subunit β-4	11q23.3	↑I_Na_	RWS
*SCN5A* [109]	Sodium channel protein type 5 subunit α	3p22.2	↑I_Na_	RWS, BrS, SSS, AS, spontaneous ventricular fibrillation, PCCD
* *ABCC9* [110]	ATP-binding cassette subfamily C member 9	12p12.1	↑I_K-ATP_	BrS, idiopathic dilated cardiomyopathy
*GATA4* [111]	Transcription factor GATA-4	8p23.1	↓Transcriptional activity	Tetralogy of fallot, etc.
*GATA5* [112]	Transcription factor GATA-5	20q13.33	↓Transcriptional activity	Tetralogy of fallot, etc.
*GATA6* [113]	Transcription factor GATA-6	18q11.2	↑Transcriptional activity	Tetralogy of fallot, etc.
*NKX2-5* [101]	Homeobox protein Nkx-2.5	5q34	↑HCN4	PCCD, hypoplastic left heart syndrome, etc.
*NKX2-6* [114]	Homeobox protein Nkx-2.6	8p21.2	Similar to NKX2-5	Tetralogy of fallot, persistent truncus arteriosus
*PITX2* [101]	Pituitary homeobox 2	4q25	↓NKX2-5	Axenfeld abnormal, Rieger abnormal, etc.
*MYL4* [115]	Myosin light chain 4	17q21.32	Ligandin	-
*TTN* [116,117]	Titin	2q31.2	-	Familial isolated dilated cardiomyopathy, early-onset myopathy with fatal cardiomyopathy, isolated arrhythmia ventricular dysplasia
*GJA5* [103]	Gap junction α-5 protein	1q21.2	Ligandin	Tetralogy of fallot
*NPPA* [105]	Natriuretic peptides A	1p36.22	Channel remodeling	AS
*NUP155* [106]	Nuclear pore complex protein Nup155	5p13.2	↓Nuclear membrane permeability	-

I_Ks_—slow delay rectifier potassium current; I_Kr_—fast delay rectifier potassium current; I_K1_—inward rectifying potassium current; I_Kur_—atrial specific potassium current; I_K-ATP_—ATP sensitive inward rectifying potassium current; I_Na_—voltage dependent sodium current; ATS—Andersen Tawil syndrome; SQTS—short QT syndrome; RWS—Romano–Ward syndrome; JLNS—Jervell–Lange–Nielsen syndrome; BrS—Brugada syndrome; PCCD—progressive cardiac conduction defect; SSS—sick sinus syndrome; AS—atrial standstill. * Candidate gene. The gene comes from HGNC.; The names of chromosome loci and loci come from OMIM. The protein comes from UniProt. Genetically related diseases come from Orphanet.

### 2.7. Idiopathic Ventricular Fibrillation (IVF)

IVF is a rare genetic arrhythmia disorder characterized by ventricular fibrillation in the absence of any structural or functional heart disease or known repolarization abnormalities [118]. The ventricular conduction system consists of cardiac Purkinje fibrocytes that have abnormal forms of transient outward current (It_o_), and too fast or too slow repolarization in cardiac cells can lead to ventricular tachyarrhythmia. Mutations in the IVF-causing gene dipeptidyl peptidase -like 6 (*DPP6*) conduce the heart to overexpress DPP6, a recognized regulator of I_to_, resulting in increased I_to_, accelerated repolarization, and facilitated IVF [119]. In addition, *SCN5A*, a gene involved in multiple sodium channel arrhythmias, is also associated with IVF [120]. Studies have shown that with *SCN5A* dysfunction mutations, sodium current is reduced, early repolarization is enhanced, and susceptibility to ventricular fibrillation is enhanced [121]. *CALM1* encoding calmodulin is also associated with IVF [122]. The mutation of *CALM1* gene affects the stability of its protein and its interaction with RyR2, then dysregulates RyR2-mediated Ca^2+^ release [123].

### 2.8. Atrial Standstill (AS)

AS is a rare arrhythmia characterized by a transient or permanent lack of atrial mechanical electrical activity and ECG findings of bradycardia, ectopic supraventricular rhythm, atrial excitability deficiency, and P-wave deletion [124]. The first found is the gene *SCN5A* inherited in an autosomal dominant way. The mutation of *SCN5A* makes the Na^+^ channel activation curve move to positive voltage, and the myocardial excitability is reduced to cause AS [125,126]. Natriuretic peptide A (*NPPA*) is found in patients with hereditary arrhythmogenic atrial cardiomyopathy combined with AS, and is inherited in an autosomal recessive manner. The specific pathogenic mechanism of NPPA is unclear, and it may be related to the progressive expansion of scar fibrosis [127,128]. Recently, lamin A/C *(LMNA*) gene mutations have also been found to be associated with AS [129].

## 3. Heart Conduction Diseases

### 3.1. Progressive Cardiac Conduction Defect (PCCD)

PCCD is an autosomal dominant inherited arrhythmia that can progress to complete heart block [130]. It is asymptomatic or presents with dyspnea, dizziness, fainting, abdominal pain, heart failure, or sudden death. The amount of sodium and the rate at which it enters the cell determine the speed at which electrical impulses are transmitted through sodium-dependent cells (muscle cells in the ventricles and atria and cells in the Schischer–Purkinje system). The mutation causes a decrease in the amount of sodium entering the cell and a decrease in the speed of conduction of the pulse, resulting in the loss of the function of the 0 phase of the action potential, which is the opening of the channel [131]. Therefore, genes that regulate sodium channels, such as *SCN5A* and *SCN1B*, encode the α and β subunits of voltage-gated sodium channels, will promote PCCD [132]. The transient receptor cationic channel encoding gene *TRPM4*, which is activated by cardiac calcium, is also considered to be a PCCD pathogenic gene, with gain-of-function mutations that lead to enhanced channel expression and membrane depolarization [133]. In addition, *NKX2-5* encoded transcription factors are essential for the maturation and maintenance of the postnatal conduction system. Studies have reported that their loss-of-function mutations can lead to the loss of DNA binding activity, atrioventricular (atV) node, and bundle and Purkinje system dysplasia, and cause a variety of cardiac conduction diseases, including PCCD, familial AF, atrial septal defects, and atrioventricular conduction block [134].

### 3.2. Congenital Heart Block (CHB)

CHB is a rare atrioventricular conduction disease characterized by atrioventricular dissociation, that is, the atrioventricular beat does not conduct to the ventricle and the heart rhythm is slow [135]. The prevalence of CHB in live births is about 1:15,000 to 20,000 [135].

Although PCCD can further develop into complete heart block with similar electrophysiological manifestations as CHB, the nature and pathogenesis of the two are different. More than half of CHB is associated with immune antibody titers of maternal association diseases [136]. In 2–5% of pregnant mothers with positive anti-Ro/SSA and/or anti-La/SSB antibodies, these antibodies attach to the placenta and damage cardiomyocytes and conduction tissues in the atrioventricular node area of susceptible fetuses via route [137]. Non-immune-mediated CHB is the result of embryonic disorders and abnormal formation of the atrioventricular node and Purkinje system, and may be associated with specific genetic markers or other pathogenic mechanisms, such as *SCN5A* mutations that impair rapid inactivation, reduce sodium current density, and thus slow myocardial conduction velocity [138]. In addition, there is also idiopathic CHB of unknown cause.

### 3.3. Lown–Ganong–Levine Syndrome (LGL)

LGL syndrome is an extremely rare conduction disorder with a prevalence of <1:10 million and is characterized by a shortened PR interval of the electrocardiogram, normal QRS waves, and atrial arrhythmia [139]. Current theories suggest that LGL syndrome may be caused by a variety of underlying causes, such as partial or complete bypass of the atrioventricular node followed by normal conduction along the bundle. However, no pathogenic genes have been reported so far.

### 3.4. Heart–Hand Syndrome (HHS)

Congenital heart malformations and upper limb malformations are often combined and are classified as HHS, the most common form of which is Holt–Oram syndrome (HOS), type II HHS (Tabatznik syndrome), type III HHS, and type IV HHS (Slovenian type) are rare [140].

HOS is an autosomal dominant syndrome characterized by upper limb malformation, mild to severe congenital septal defect, and conduction defects (e.g., paroxysmal atrial fibrillation, atrioventricular block). More than 85% of HOS can be explained by mutations in the T-box transcription factor 5 gene (*TBX5*) located on the long arm of chromosome 12 (12q24.1) [141]. The *TBX5* gene encodes the TBX5 protein, a transcription factor that regulates the expression of other genes in the heart and limbs during development. TBX5 and homologous domain transcription factor Nkx2-5 have direct and synergistic trans-activation of atrial natriuretic peptide and Cx40 promoter [142]. Combined with the important role of Nkx2-5 in the cardiac conduction system described above, it is not difficult to understand the pathogenic role of *TBX5* mutations in HOS.

Type II HHS is characterized by congenital arrhythmias and type D brachymelia involving the upper limbs, and type III HHS is characterized by cardiac conduction disorders and type C brachymelia accumulating in the hands and feet, seen in newborns and infants. No pathogenic genes have been reported in either of them.

Adult onset of type IV HHS is characterized by progressive sinoatrial and atrioventricular conduction disease and atrial and ventricular tachyarrhythmia, which can lead to sudden death. It was found that type IV HHS is caused by lamin-coding gene *LMNA* mutation and is inherited in an autosomal dominant manner. *LMNA* genes encode laminin A and C through alternate splicing, which can lead to nuclear membrane abnormalities when mutated. *LMNA* is also the most commonly mutated gene in dilated cardiomyopathy with conduction system disease, and it behaves very similarly to type IV HHS [143]. In addition, *LMNA* mutations also affect rhabdomyolysis.

### 3.5. Other Heart Conduction Disorders

Familial atrial tachyarrhythmia subfascicular heart conduction disease is an autosomal dominant genetic disease caused by TNNI3 interacting kinase (*TNNI3K*) mutation, with a prevalence of <1:1,000,000. *TNNI3K* encodes a heart-specific kinase known to regulate heart conduction and myocardial function, and when mutated, the protein aggregates and reduces the concentration or bioavailability of the protein in cardiomyocytes, which may be the basis for the development of inherited heart disease [144].

## 4. Other Hereditary Arrhythmia Disorders

A single gene can participate in multiple biological pathways and play a variety of pathophysiological functions. Some of the genes that cause arrhythmia can also cause other symptoms or diseases, such as cardiomyopathy, limb deformity, intellectual disability, deafness, and so on. These genes and the diseases they cause are discussed below (Table 5).

Chronic atrial and intestinal dysrhythmia syndrome (CAID) is characterized by chronic atrial and intestinal dysrhythmia with a prevalence of <1:10 million [145]. The cohesive protein complex encoded by Shugoshin-1 *(SGO1)* plays a central role in mitosis, while a single homozygous mutation of *SGO1* leads to a shortened cell cycle, high senescence rate, transforming growth factor, and transforming growth factor in fibroblasts. TGF-β signal transduction is enhanced and its downstream product SMAD2/3 phosphorylation levels increase, with translocation from nucleus to cytoplasm [146]. On the other hand, chronic TGF-β activation leads to intestinal ganglion hyperplasia, mislocalization of Cajal cells, structural rupture of smooth muscle fibers, extensive fibrosis, and thinning of the intestinal smooth muscle layer, leading to CAID syndrome [145]. Recent research has found that mutations in the gene *SGO1* (p. Lys23Glu), which codes for the polymeric protein Shugoshin-1, can lead to severe arrhythmias caused by central atrioventricular node dysfunction, and debilitating gastrointestinal motor disorders. Shugoshin-1 interacts directly with HCN4 to promote and stabilize cardiac pacing. Mutations p. Lys23Glu impair the interaction between Shugoshin-1 and HCN4, and inhibit comical current and rhythmic activity in cardiomyocytes of patients with induced pluripotent stem cell-derived CAID [147].

Histiocytoid cardiomyopathy (HCM) is characterized by hypertrophy of the heart, severe arrhythmia, or sudden death, and the presence of histiocytoid cells in the heart muscle [148]. Current genetic analysis has shown that there are two candidate genes for this disease: *MT-CYB*, which encodes mitochondrial cytopigment b [149], and *NDUFB11* [150], which encodes the NADH dehydrogenase β subunit of the mitochondrial membrane respiratory chain. Mutations may reduce the stability of the mitochondrial complex and affect mitochondrial energy production, thus promoting the disease.

Blood vessel epicardial substance (*BVES*) associated limb girdle muscular dystrophy (LGMD) is characterized by atrioventricular block, resulting in repeated syncopal episodes, elevated creatine kinase serum levels, and slow-progressing proximal limb skeletal muscle weakness and atrophy in adults [151,152]. The mutations decrease cAMP affinity, impair membrane transport, and increase action potential hyperpolarization and rise rate, which may be related to the arrhythmia of LGMD [153].

*GNB5* encodes G protein β subunit 5, which is involved in inhibiting G protein signal transduction. *GNB5* mutations can cause intellectual disability arrhythmic syndrome, characterized by heart rate disturbance, eye disease, intellectual disability, pathological gastroesophageal reflux, low eye pressure, and seizures. Individuals with lose-function alleles have more severe symptoms, while individuals with heterozygotes containing missense mutations have a less severe clinical phenotype [154].

X-linked intellectual disability/cardiac hypertrophy/congestive heart failure syndrome is associated with chloride intracellular channel 2 (*CLIC2*) mutations and is characterized by X-linked intellectual disability, AF, cardiac hypertrophy, congestive heart failure, epilepsy, and certain physical features. The mutation stimulates RyR channel activity and prolongs the time the channel remains open, potentially amplifying Ca^2+^ signaling that depends on RyR channel activity. Overactivity of RyR in heart muscle, skeletal muscle cells, and nerve cells leads to abnormal heart function and triggers the release of postsynaptic pathways and neurotransmitters [155].

Transport and Golgi organization 2 homolog (*TANGO2*) encodes a protein involved in the redistribution of the Golgi membrane to the endoplasmic reticulum, and its biallelic cutoff mutation causes defective mitochondrial fatty acid oxidation, leading to recurrent metabolic myogenic brain crisis/rhabdomyolysis/arrhythmia/intellectual disability syndrome [156].

Progressive sensorineural hearing loss/hypertrophic cardiomyopathy syndrome is associated with myosin VI (*MYO6*) mutations and is characterized by sensorineural hearing loss, prolonged QT interval, and mild cardiac hypertrophy. Non-muscle myosin encoded by *MYO6* has motor activity on the negative end of the actin filament. The variants may have a negative effect on dimerization, impair the motor function of the dimer, and affect the formation of cytoskeleton and cell motor function, resulting in pathological changes of hypertrophic cardiomyopathy [157].

Sinoatrial node dysfunction and deafness (SANDD) presents as deafness without vestibular dysfunction, with sinoatrial node dysfunction, bradycardia, increased resting heart rate variability, and paroxysmal syncope [158]. *CACNA1D* encodes the voltage-gated L-type calcium channel pore-forming α1D subunit, which is expressed in neurons and neuroendocrine cells. The pathogenic mutation of *CACNA1D*, on the one hand, makes the L-type current of the hair cells in the cochlea weaken or even disappear, and the hair cells become denatured, which can cause deafness; on the other hand, reducing the threshold of channel activation, mechanical deceleration of inactivity, and controlling the depolarization of sinoatrial node during diastolic period can lead to sinoatrial node dysfunction [158,159].

There are also a small number of rare inherited heart diseases, which have not been shown to be linked to genetics, for example, short thumb syndrome and short interphase tip torsion ventricular tachycardia syndrome. In addition, at present, the causes of rare non-hereditary arrhythmias are unclear, including idiopathic neonatal atrial flutter, persistent infant ventricular tachycardia, multifocal atrial tachycardia, etc.

**Table 5:** Pathogenic genes and inheritance modes of other hereditary arrhythmia disorders.

Disease	Hereditary Mode	Gene
Chronic atrial and intestinal dysrhythmia syndrome (CAID)	AD	*SGOL1*
Histiocytoid cardiomyopathy (HCM)	XD/AR	*MT-CYB*, *NDUFB11*
Limb girdle muscular dystrophy (LGMD)	AR	*BVES*
Intellectual disability/arrhythmic syndrome	AR	*GNB5*
Intellectual disability/cardiac hypertrophy/congestive heart failure syndrome	XR	*CLIC2*
Recurrent metabolic myogenic brain crisis/rhabdomyolysis/arrhythmia/intellectual disability syndrome	AR	*TANGO2*
Progressive sensorineural hearing loss/hypertrophic cardiomyopathy syndrome	AD	*MYO6*
Sinoatrial node dysfunction and deafness (SANDD)	AR	*CACNA1D*

AD—autosomal dominant inheritance; AR—autosomal recessive inheritance; XD—X-linked dominant inheritance; DR—X-linked recessive inheritance.

## 5. Genome-Wide Association Study (GWAS) of Hereditary Arrhythmia Diseases

With the continuous in-depth study of genetics, diseases are not only caused by rare mutations in a single gene locus but also by the accumulation of mutations in many different genes. A growing body of evidence suggests that hereditary arrhythmia diseases may be a polygenic disorder rather than a simple Mendelian monogenic disorder. A small subset of genotype-negative patients may have an unknown Mendelian defect, but it is also possible that a subset of these patients may have a more complex genetic pattern, perhaps caused by the accumulation of multiple variants. GWAS is a genome-wide method of investigating the association between genetic variation and a trait or disease. With the continuous development of high-throughput sequencing technology, GWAS has achieved remarkable results in the past decade, identifying many genetic susceptibility sites for complex diseases [160]. Because hereditary arrhythmia diseases are relatively rare, it is difficult to collect a large number of cases, and there are relatively few GWAS studies on these diseases. Current GWAS studies have identified three LQTS susceptibility sites near *NOS1AP*, *KCNQ1*, and *KLF12*. The association of the *KCNQ1* genetic variants suggests that common variants in these genes work together with rare variants in mediating disease susceptibility [161]. The latest GWAS in a Japanese population identified 1 new BrS genetic susceptibility locus near *ZSCAN20* and 17 susceptibility loci were identified by cross-ancestry meta-analysis, 6 of which were newly identified [162]. In addition, the researchers identified *DNAJC6* as a novel susceptibility gene for CHB through GWAS, and the decreased cardiac expression of *DNAJC6* was associated with the disease risk genotype [163]. These evidences suggest that common genetic variants also play an important role in hereditary arrhythmia diseases, and the role of these common predisposition variants should not be ignored during genetic screening.

## 6. Conclusions

As one of the most important causes of sudden cardiac death, hereditary arrhythmia not only poses a serious threat to the life of patients but also poses a great challenge to clinicians, so it is of great significance to conduct in-depth research on it. The study of hereditary arrhythmias has made significant progress in the past few decades, providing important clues for us to better understand their pathogenesis and clinical characteristics.

At present, a number of pathogenic genes have been identified through various technical means, and the future research direction should further explore the relationship between pathogenic genes and pathogenesis. With the continuous development of gene sequencing technology, we are expected to identify more disease-causing genes related to hereditary arrhythmias more accurately in order to reveal the mechanism of their influence on cardiac electrophysiological abnormalities. A deeper understanding of the functions and interactions of these pathogenic genes will help reveal the complex pathogenesis network of arrhythmias. Further, based on a better understanding of the pathogenesis of hereditary arrhythmias, we hope to provide more precise strategies for their prevention and treatment. Through the intervention of specific pathogenic genes, the electrophysiological function of the heart can be targeted to reduce or prevent the occurrence of arrhythmia. In addition, the application of emerging fields such as drug development, gene editing technology, and stem cell therapy is also expected to bring new breakthroughs in the treatment of hereditary arrhythmia. According to different pathogenic genes, the classification treatment of arrhythmia diseases has become the first classic example of individualized genetic diagnosis and treatment in the medical field. The combination of modern life science and medicine has changed the traditional diagnosis and treatment mode of detection, treatment, and new drug discovery in the past, and can determine more detailed disease prevention strategies and drug treatment plans according to the genetic characteristics and molecular pathogenesis of different groups or individuals, and gradually realize personalized treatment.

To sum up, remarkable progress has been made in the field of genetic arrhythmia, but there are still many problems and challenges to be solved. Through continuous and in-depth research, we are expected to reveal more pathogenic mechanisms, pathogenesis, and treatment methods, and provide more powerful support for reducing the risks associated with arrhythmia and safeguarding the health of patients.
